# An Evaluation of a Women’s Clinic: The Healthcare and Learning Project of the Functional Unit for Women with Schizophrenia

**DOI:** 10.3390/healthcare12151483

**Published:** 2024-07-26

**Authors:** Alexandre González-Rodríguez, Mentxu Natividad, Bruma Palacios-Hernández, Rosa Ayesa-Arriola, Jesús Cobo, José A. Monreal

**Affiliations:** 1 Department of Mental Health, Mutua Terrassa University Hospital, Fundació Docència i Recerca Mutua Terrassa, University of Barcelona, 08221 Terrassa, Spain; mnatividad@mutuaterrassa.cat (M.N.); jamonreal@mutuaterrassa.cat (J.A.M.); 2 Perinatal Mental Health Research Laboratory, Center for Transdisciplinary Research in Psychology (CITPsi), Autonomous University of the State of Morelos, Cuernavaca 62350, Mexico; bruma.palacios@uaem.mx; 3 Department of Psychiatry, Marqués de Valdecilla University Hospital, Instituto de Investigación Sanitaria Valdecilla (IDIVAL), School of Medicine, University of Cantabria, 39005 Santander, Spain; rayesa@idival.org; 4 Faculty of Psychology, National University of Distance Education (UNED), 39008 Santander, Spain; 5 Department of Mental Health, Hospital Universitari Parc Taulí, 1 Parc Taulí, 08208 Sabadell, Spain; jcobo@tauli.cat; 6 Department of Psychiatry and Forensic Medicine, Universitat Autònoma de Barcelona, Plaça Cívica, 08193 Bellaterra, Spain; 7 Institut de Neurociències, Universitat Autònoma de Barcelona (UAB), 08221 Terrassa, Spain

**Keywords:** learning techniques, healthcare, innovation, schizophrenia, women

## Abstract

Gender differences exist in mental and physical health in schizophrenia, and healthcare education is part of the associated clinical approach. The main goal of the present paper is to describe a women’s clinic for schizophrenia and carry out a narrative review about innovative healthcare and learning strategies in the context of women who suffer from schizophrenia, and to discuss innovative strategies for both healthcare and learning projects to be applied in this context. Observing the development of our unit, four clear innovation phases can be distinguished: the generation of new ideas (clinical and social needs), strategic planning (five observatories), the execution of these strategies (observatories/teams/interventions) and feedback, iteration and scaling. We found that the observatory for morbi-mortality adopted a retroactive proactive approach, and the observatory for hyperprolactinemia was proactive and deliberate. We describe the innovation aspects, both clinical and educational, as incremental. There was one exception, the introduction of a social exclusion and discrimination observatory, that from our perspective, was not gradual, but transformative. Future learning projects should include the role of social sciences and humanities and new technologies. Our pilot project gave us the opportunity to apply new learning methods to a relatively neglected field of care.

## 1. Introduction

### 1.1. Health and Social Needs of Women with Schizophrenia

A high amount of scientific literature supports the fact that many gender differences exist in schizophrenia in the epidemiological, preclinical, and clinical aspects [1]. In fact, women with schizophrenia show a later age at onset of the illness compared to men, and a second peak of incidence at the time of menopause [2]. These gender differences in epidemiological aspects add to the clinical differences shown between men and women regarding clinical symptoms and therapeutic response. While men with schizophrenia are more likely to present negative and cognitive symptoms, women more frequently develop positive and affective symptoms [3]. Women with schizophrenia have a better therapeutic response than men, at least when comparing premenopausal women with men [4]. Social functioning appears to be better in women than in men during the women’s reproductive years, and the overall prognosis have been found to be better in women [5]. At the time of menopause, a period characterized by a drop in estrogen levels, women with schizophrenia experience the worsening of psychotic symptoms, impairment in cognitive domains, and higher social needs compared to women with schizophrenia in the reproductive period [6].

Gender differences in schizophrenia are not only related to mental health symptoms [7,8]. Differences in physical health and psychosocial risk factors have also been reported in the scientific literature [7,8].

The morbi-mortality risk is increased in schizophrenia [9,10,11]. Women suffer from more severe negative consequences compared to men, as many of the medical comorbidities are frequently later recognized [4,12]. Of all the medical comorbidities, cardiovascular risk and respiratory diseases are the most commonly found, followed by cancer mortality [13,14]. In line with these well-known factors, a retrospective cohort study investigated cancer mortality and morbidity in patients with schizophrenia [15]. Women had a higher mortality risk compared to men, with respect to different cancer types. Furthermore, the mortality rates of breast cancer in women with schizophrenia are higher than expected, probably due to a delayed diagnosis. Breast cancer screening completion seems to be lower among women with schizophrenia compared to women without schizophrenia [16]. In summary, lifestyle and illness behaviors are associated with the overall morbi-mortality risk in women with schizophrenia. Innovative specific interventions on health needs may reduce these risks and increase the life expectancy of women.

Furthermore, it should be noted that social determinants of mental health are factors that are relevant to the development of schizophrenia and affect health through biological pathways [17]. The social determinants of mental health are environmental, structural and social factors that impact the clinical expression and prognosis of mental disorders and may also include health inequities. They are also the conditions in which people are born, live and work, and increase health disparities. Social risk factors affect men and women; however, these have been demonstrated to exert a more negative effect on women. Women with schizophrenia are negatively affected by discrimination, social exclusion, social isolation, urbanicity, inadequate housing, poor socioeconomic status and childhood and adult trauma [18,19]. Innovative interventions with the co-participation of patients, their families and communities should be designed to better engage patients and to focus on the real health, social and economic needs of themselves and their offspring. To the best of our knowledge, few studies have reported the inclusion of participative interventions in the design of clinical care of women with schizophrenia and related disorders [20,21]. Integrative programs are highly recommended.

### 1.2. Innovation in Mental Health Care

Innovation in mental health care means the development or delivery of new solutions for the prevention, treatment, or rehabilitation of difficult mental health conditions. During the last decade, personalized medicine has provided a framework that helps advance such innovation strategies [22]. It includes the formation of large scientific collaborations and the building of digital tools to improve clinical practice. Personalized medicine has substantially influenced mental health care. A recent paper highlighted the increased progress in the development of telemedicine and virtual care during the COVID-19 pandemic, and the increase in virtual resources to collaborate with other mental health professionals and partners [22]. Sharing knowledge and optimization of technology to improve healthcare and learning outcomes result from the influence of the personalization of care through new strategies. For instance, virtual tools to share the most recent treatment to treat mental disorders, or virtual whiteboards to share ideas and design projects with other mental health professionals [23].

Also included are collaborations with primary care services [24]. A novel program, whether clinical or educational, should be able to demonstrate clear benefits when compared to the status quo [25]. The value of innovation in teaching and learning at the university level is immeasurable since the future of healthcare depends on the thorough and up-to-date preparation of today’s students [26]. Psychiatric illnesses are acknowledged to be multidetermined; the basic causes remain unknown. This means that innovation in research, in teaching, and in the delivery of care is particularly important in the field of mental health. Ethical considerations should be included in the design and implementation of health and learning strategies, such as the use of artificial intelligence, big data and technologies like the Internet of Things (IoT).

Another promising field, revealed by progress in genetic knowledge, is personalized care—the increasing ability to address the particularities of individual patients rather than assuming that the standard treatment of a specific diagnosis will be equally successful for all [27]. As a starting point, we have learned that age and sex/gender are important determinants of effective treatment [28]. For instance, the many epidemiological, clinical, and response-to-treatment differences between men and women affected by schizophrenia [29,30,31,32] have led to the establishment of treatment programs exclusively for women [33]. Seeman and Cohen [34], on the premise that women’s needs (reproductive, cognitive, familial, pharmacologic, safety, interpersonal and comorbid) significantly differed from those of men and could be more effectively treated in a specialized unit, set the first such outpatient unit in 1995.

### 1.3. Innovation in Healthcare Learning

Interventions to improve healthcare include those focusing on the interaction of professionals and patients with the aim of improving self-care. The clinical benefits of lifestyle interventions have been demonstrated in many ways [35]. Physicians, nurses and other healthcare professionals should promote patient education and engagement to improve self-health management and responsibility in healthcare utilization. Personalized patient education is needed, including strategies for patient empowerment [36]. Educational materials developed by healthcare providers should be incorporated in the electronic health record system, for instance, by using a patient portal to help patients in terms of healthcare education [36]. A high amount of literature supports the notion that the development of patient education programs requires the training of professionals and financial support for the healthcare institutions and organizations [37]. These programs should be innovative and include an active role for patients in their healthcare.

In the context of people suffering from severe mental illness, health education is important to promote engagement and motivation for changing lifestyles. Several barriers and facilitators have been described [38]. Barriers to the active role of patients in health education are (1) the use of biomedical rationales for changing behaviors; (2) avoiding asking about their needs and preferences; and (3) using one-way communication without enough time for shared reflection. Traditional education techniques have been frequently used. To engage patients in healthcare education activities, communication tools and skills should be facilitated by healthcare professionals.

Recently, some authors pointed out that innovation in mental health care education should include the extension of collaborations and building of digital tools, for instance, the patient portal [39]. Personalized patient education is crucial, and healthcare professionals can effectively promote patient education by using psychological approaches focused on working cognitive skills and motivation. Promoting engagement and empowering patients to participate in their own healthcare processes is one of the main goals of patient education [36]. For instance, some authors have reported that educating a patient suffering from osteopenia on continuing or starting vitamin D supplementation, participating in exercise groups and following a healthy diet are examples of interventions with clinical benefits [39].

However, to date, very few works have been focused on the development of patient education programs to treat women suffering from schizophrenia.

### 1.4. Aims

The main aim of this paper is to describe a women’s clinic for schizophrenia and related disorders, to analyze potential areas for the innovation of these clinics, and to discuss a health and learning project for patients. This paper will deal with the following points:

1. The description of the health project, including the model of observatories, vigilance teams, and specialized interventions;
2. To review evidence of innovative ideas in healthcare for women with schizophrenia and learning projects;
3. To discuss women’s clinic healthcare and learning projects in terms of innovation;
4. To propose future innovative strategies for the women’s clinic.

## 2. Methods

### 2.1. Description of the Functional Unit for Women with Schizophrenia

The Mutua Terrassa Functional Unit for Women with schizophrenia in Terrassa, Barcelona, Spain, was inaugurated in January 2023 as a pilot project in the context of the Adult Community Mental Health Services [33]. Initially, the structure and management of the unit was divided into two—a science corner and a social corner (see Table 1). The science corner mainly consisted of five working “observatories” of health whose goals were to improve the clinical care of women with schizophrenia.

We will describe the components of the five observatories of health and social risk factors and the development of the unit.

### 2.2. Narrative Review on Innovation in Healthcare and Learning Projects for Women with Schizophrenia

#### 2.2.1. Search Strategy

We conducted a narrative review using the PubMed database from inception to July 2024. PubMed was used as it captures most of the medical literature on women’s mental health. The following search terms were used: (innovation OR innovative OR reactive OR proactive OR incremental OR radical OR transformative OR STEAM OR SHAPE OR “design thinking”) AND schizophrenia AND women.

The search was combined with a Google Scholar search to gain access to other papers in the field of innovation in healthcare and learning projects. Other papers considered of relevance in the field of learning or education were included.

#### 2.2.2. Inclusion and Exclusion Criteria

Papers were included if they referred to innovation strategies to improve healthcare and learning projects. Papers including concepts of proactive and reactive approaches in innovation, and radical, transformative and incremental approaches were selected, to discuss the implementation and design in terms of innovative practices.

#### 2.2.3. Data Collection and Extraction

The PubMed database was first searched for relevant titles and abstracts. Other sources were also searched to find additional papers of interest in the field of innovation. Three-hundred records were found with our initial search strategy.

### 2.3. Analysis and Discussion of the Healthcare and Learning Projects in Terms of Innovation

The results of the narrative review about the innovative approaches to healthcare and learning projects will be applied to the 5 observatories of health.

Additionally, the learning or educational programs for patients have been programmed. The group of substance use disorders, physical exercise, nutrition and dietary recommendations and discrimination/social exclusion groups were designed according to the 6 levels of cognitive processes.

## 3. Results

### 3.1. Description of the Functional Unit for Women with Schizophrenia in Terrassa

The Functional Unit for Women with Schizophrenia of the Mutua Terrassa University Hospital was inspired by previous clinical projects. The main source of inspiration was a service for women with schizophrenia that was established at the Clarke Institute of Psychiatry at the University of Toronto, in 1995 [34]. The original project was led by Professor Mary V. Seeman, who was a Professor of Psychiatry at the University of Toronto. This unit combined inpatient and outpatient care and community services and included patient and family assessment. If necessary, the clinical team offered home-based outreach services and substance use counseling. Women with schizophrenia who were mothers or caring for children received parenting training. Psychotherapy groups, sex education and self-protection strategies in urban environments were offered to prevent victimization. Linkages between the unit and fitness programs and leisure activities were programmed in the unit.

In our Functional Unit for Women with Schizophrenia, a total of 34 professionals from the two adult community mental health services (Terrassa and Sant Cugat) were involved. Further details are described elsewhere [9,33]. All professionals are employed part-time by the unit. That means they spent part of their time working in the unit, and the remaining time covering the clinical care of men and women with other severe mental illnesses.

The two adult mental health services amalgamated in the functional unit cover the clinical and social care of around 390 women with schizophrenia and other related disorders. The currently offered programs has been previously described [9,33]. Target women (this is the name that refers to women fulfilling the criteria in the specialized unit) are defined as women with schizophrenia that need specific interventions that are not covered by the routine clinical practice in community mental health clinics. These women are assigned to one of the five observatories and then included into the unit. When the women stop needing these specific interventions, they are returned to the clinical care of the adult mental health community services.

Data collection tools may include (1) an assessment of the mental health diagnosis according to the current statistical manual of mental disorders, (2) an assessment of adherence to the community mental health clinics and professionals in charge of the cases, (3) an evaluation of the medical comorbidities and treatments, (4) an assessment of the prolactin levels and evaluation of treatment plans, (5) a prescription of benzodiazepines, the use of alcohol and the presence of other substance use disorders, (6) an assessment of the social determinants of mental health, migration processes and cultural background of the patients, (7) a prescription of antipsychotics and antidepressants, (8) an assessment of safety concerns (e.g., risk of falls).

Evaluations may include the development of process and outcome indicators. Process indicators are designed to assess the status of a task and progress compared to the established objectives. For instance, a process of the unit may include the identification of a number of women with schizophrenia with specific clinical and social needs. An example of a process indicator would be the description of a number or percentage of women with schizophrenia suffering from moderate to severe hyperprolactinemia to be included in a specific intervention of the unit. Every year, the authors will design process indicators with an evaluation of the results from every trimester. Moreover, outcome indicators will be designed to measure the results of an intervention. They refer more to the objectives of the unit. For example, a reduction in prolactin levels of at least 30% may be an outcome indicator referring to the objective of an intervention.

Our unit was designed to have five main observatories that served as working groups based on five research and clinical problems: (1) somatic morbi-mortality, (2) hyperprolactinemia, (3) substance use disorders, (4) social exclusion and discrimination and (5) prescription and drug safety.

The first observatory, dealing with morbi-mortality, reviews and analyzes in monthly staff meetings all the deaths of the women to determine how they could have been prevented. For instance, we reviewed the prevalence of sleep apnea and cardiovascular risk factors in the women of our unit. The clinical staff monitor medical comorbidities and design specific interventions to reduce mortality and to eliminate risk factors that affect function and quality of life. An analysis of case reports is proposed at the monthly meeting to illustrate specific medical problems that can be applied to women who suffer from schizophrenia. Preparing material beforehand allows for more knowledgeable and productive discussions.

The goal of the observatory for hyperprolactinemia is to monitor, prevent and treat antipsychotic-induced hyperprolactinemia. Reviews of reports of prolactin tests, physical exams and personal histories are read out at the observatory meetings to determine who is at risk, and solutions are proposed. Clinical guidelines are reviewed, and one person is selected to report on the latest clinical recommendations in the field.

The observatory for substance use disorders, in its monthly meetings, reviews substance use in the unit, particularly benzodiazepine dependence, opioid consumption and alcohol use. A principal aim of this observatory is to reduce the prescription of benzodiazepines, which is considered too frequent. Clinical guidelines are reviewed, and an addictions specialist is called in to make treatment suggestions.

The observatory for social exclusion and discrimination was initiated to identify the health and social needs of women at risk of discrimination and exclusion, mainly focusing on the optimum clinical care of immigrant women, which represents an important sector in Catalonia, Spain. Cooperative working strategies are applied to propose specific interventions such as focus groups to explore the needs and expectations of patients who appear to be socially excluded. Individual case studies are presented to propose solutions to problems deriving from discrimination, stigma and bias.

The observatory for prescription and drug safety monitors the effectiveness and tolerability of prescribed drugs, with a special emphasis on drug resistance. Clozapine prescription for treatment-resistant schizophrenia is often proposed after a discussion of the risks and benefits. Interventions such as drug levels are often recommended, carried out, and the results are discussed.

These five working observatories all started at the time of inauguration of the unit.

At the end of the first year, the Unit for Women with Schizophrenia introduced a sixth focus, the observatory for sustainability and resource optimization, which deals with the United Nations’ 17 Sustainable Development Goals aimed at ending poverty, reducing inequities, and addressing climate change [40]. A second objective was to optimize the quality and efficiency of the currently offered programs and to plan new transdisciplinary strategies of care. This last observatory is still in development. Table 2 summarizes the main characteristics of the five original observatories and the planned evaluation methods for each observatory.

### 3.2. Designing the Four Phases of Healthcare Innovation

The Unit for Women with Schizophrenia is innovative from the perspective of healthcare delivery and from a teaching/learning perspective focused on patients.

It selects a specific, relatively neglected, target of intervention—women with schizophrenia. It chooses specific targets of care in this population that differ between men and women.

The first observatory, for somatic morbi-mortality, concentrates on morbidity associated with schizophrenia for which women are most at risk. It studies lifestyle, screening and monitoring strategies for early prevention and consults with appropriate specialists for beginning early effective treatment and assuring its maintenance. The observatory for hyperprolactinemia considers the fact that women’s baseline prolactin levels are higher than men’s and that they are more prone to anxiety, which, itself, raises prolactin levels. The symptoms of high prolactin (acne, hirsutism) affect women’s quality of life and amenorrhea, breast swelling and galactorrhea induce delusions of pregnancy [41]. By lowering estrogen levels, high prolactin induces osteoporosis and lowers the efficacy of certain antipsychotics. Hyperprolactinemia is also suspected of heightening the risk of breast cancer [42]. The aim is to adjust medications toward those that are least likely to cause hyperprolactinemia and to consult with specialists when necessary [43].

The observatory for substance use disorders monitors and intervenes, particularly with respect to benzodiazepine use and alcohol use disorders. While men are more likely than women to suffer from substance abuse disorders, the sequelae of these disorders progress more quickly in women [44]. Women with substance use disorders are themselves at high risk of victimization and consequent trauma, often requiring trauma interventions [45]. A reduction in benzodiazepine prescription is also an objective of this clinical observatory that implies a reduction in the risk of adverse events.

The observatory for social exclusion and discrimination focuses on the social needs of marginalized women, ethnically distinct women, women with physical disabilities, and particularly new immigrants. Refugee women are at particular risk because of the trauma they experience prior to leaving their countries, especially due to the high frequency of gender-based violence, the duress of the migration journey, and the difficulties of acculturation in the host country [46]. They experience different risks to refugee men because of the dangers of sexual exploitation during migration and the concerns for children and relatives left behind [47]. They require cultural awareness on the part of care givers because psychiatric symptoms may be heavily stigmatized in their home countries [48,49]. This approach may improve the access and participation of migrant women in our mental health services.

The observatory for prescription and drug safety is critical because the pharmacokinetics and pharmacodynamics of therapeutic drugs for schizophrenia differ in women and men. Effective doses differ and the tolerability of side effects differ [50]. Many women require dose adjustments over the menstrual cycle, during pregnancy and the postpartum, and during the lead up to menopause, as well as post menopause [51]. Antipsychotic combinations have been reduced, a fact that may have positively impacted on the tolerability of side effects and safety of women with schizophrenia.

Table 3 summarizes the four main phases of a successful innovation project. This is divided into innovations affecting the direct implementation of healthcare interventions for patients and innovations affecting learning interventions for patients.

### 3.3. Analyzing the Innovative Healthcare Approach in the Unit for Women with Schizophrenia

In our review, we found that innovation healthcare projects can be defined according to the nature of the strategy into reactive and proactive approaches, incremental, radical or transformative approaches, and transdisciplinary approaches if they are integrated in sciences or humanities.

#### 3.3.1. Reactive and Proactive Approaches

Reactive management refers to strategies used to respond to unanticipated events, the aim being to arrive at a solution to a particular situation. In other words, a reactive strategy is a response to an event, taking hyperprolactinemia as an example, that has already occurred [52]. In contrast, proactive management is used to anticipate and prevent unwanted events from happening. The goal here is to reduce or altogether avoid anticipated negative events. In our unit for women with schizophrenia, the observatory for hyperprolactinemia was designed to screen and prevent (proactive), monitor (reactive) and treat (reactive) hyperprolactinemia in patients on antipsychotic medication because hyperprolactinemia has short-term and long-term consequences on physical health [52]. The focus was on proactive strategies to anticipate and prevent the development of hyperprolactinemia to prevent somatic and psychological consequences. Innovative learning strategies (case presentations, patient interviews, scientific experiments showing how a reduction in dopamine caused by antipsychotics causes an elevation in prolactin levels, which, in turn, lowers estrogen levels, and how this course of events is enhanced in the presence of stress) help students and staff understand mechanisms and apply them to their patients [33,50]. The proactive approach of the observatory for hyperprolactinemia may prevent polypharmacy, which is frequent in the treatment schizophrenia, as well as extrapyramidal effects and the risk of falls. In many cases, the strategies used to reduce hyperprolactinemia are centered on the reduction in the doses and the optimization of antipsychotic medications. In the long term, lowering prolactin levels has been shown to reduce the risk of osteoporosis, and by implication, the risk of falls.

In the somatic morbi-mortality observatory, the monthly staff meetings help to identify clinical symptoms and to propose evidence-based treatment (reactive) to prevent further risks. There is also a retrospective aspect where causes of death are analyzed, and consideration is given to how they might have been prevented. Cardiovascular and respiratory mortality is often preventable if risks, both from schizophrenia-related habits and antipsychotic-induced side effects, are addressed early [53]. This would constitute retroactive proactivity. Physical exercise groups are proactive strategies that reduce the risk of obesity and cardiovascular problems, which require new learning strategies.

#### 3.3.2. Incremental, Radical and Transformative Approaches

There are other ways of describing innovative learning approaches, such as dividing them into incremental, radical and transformative [54,55]. Incremental innovation refers to gradual and iterative improvements or upgrades to standard practice with the aim of incorporating small minor improvements via feedback from patients or teachers or by including in one’s repertoire the results of new research. In all the observatories, the sharing of information at monthly meetings moved the learning curve incrementally forward. Medical advances, usually, move slowly because the effectiveness of new approaches must be checked and rechecked and the adverse effects of change need permanent evaluation [54,55].

Radical or disruptive innovation means the relatively quick adoption of a new process or a new treatment, usually dependent on the use of new technology. A recent systematic review investigated barriers and facilitators of radical innovations in healthcare [55]. The main facilitators of this form of innovation are a supportive culture and well- educated, highly trained, and knowledgeable users. Barriers to transformational change are the lack of human, financial and material resources. In our unit this approach has not been implemented.

Transformative approaches to innovation incorporate the patient or user perspective. In fact, transformative projects involve patients in clinical decision making. In the Functional Unit for Women with Schizophrenia, women are asked for their perspectives on the type of intervention they would find most helpful. This is especially important for women from backgrounds and health traditions different from those in the majority culture [56]. Religious rites, for instance, may be important to their healing [57]. Patients’ creative ideas should also be incorporated when planning transformative projects [58,59]. In our experience, this transformative approach helped the patients to improve their access and participation in the clinical decision making processes and to increase their trust to our services.

#### 3.3.3. Transdisciplinary and Cross-Sectional Approaches

##### STEAM Projects

STEAM projects are modern learning methods focused on holistic thinking that incorporate aspects of science, technology, engineering, art, and mathematics (STEAM). Learning experiences are highly influenced today by the rapid advance of technologies such as artificial intelligence (AI), which can be applied to both healthcare and learning [60]. To prepare professionals for the new opportunities and challenges of the future, STEAM projects are crucial. For instance, the observatory for hyperprolactinemia aims for the prevention, early detection, monitoring, and treatment of antipsychotic-induced hyperprolactinemia. The use of gamification techniques increases the motivation of both staff and students to learn about and apply this knowledge in the clinic. Gamification refers to the introduction of basic game elements (rewards such as points, levels, badges, competitive goals) into learning tasks. We are beginning to use gaming techniques to introduce competition and motivation among our learners. We all accumulate points and knowledge cards and keep them in a didactic suitcase containing items that stimulate learning [61].

The inclusion of STEAM programs in future learning initiatives is based on the need to highlight the connections between science and culture [61]. The Functional Unit for Women with Schizophrenia could generate a group formed by multidisciplinary professionals and patients to develop several projects assigned to the clinical observatories. The main goal of the STEAM projects is improving physical health, caring for aging patients, increasing social connections, and working on the knowledge of different cultures of migrant women with schizophrenia. The future educational strategies of the clinic may be based on, at least, five projects: (1) physical exercise, (2) nutrition, (3) migration, (4) culture, and (5) the risk of falls or overmedication. Mindfulness and other non-pharmacological strategies may be included to reduce stress for both professionals and patients.

##### SHAPE Projects

SHAPE projects refer to ones addressing social sciences, humanities, arts and the economy. Most such projects take a positive view of what can be accomplished in the future [62]. These innovation projects aim to develop strategies with a mainly social and environmental impact [63]. Our new observatory for sustainability and resource optimization falls into this category [40]. We are developing targets, actions and indicators related to the other observatories. For instance, the vigilance team of the observatory for somatic morbi-mortality is studying dietary habits and reviewing nutrition recommendations for women with schizophrenia who have trouble affording healthy food for themselves and their families [64].

The inclusion of SHAPE programs in learning processes may increase the interaction between the social sciences, the humanities and the economy/environment. The observatory for sustainable development and resource optimization aims to work, by means of the education of both professionals and patients, on sustainability challenges described by the 17 Sustainable Development Goals adopted by all United Nations Member States in 2015. Physical and mental health are both influenced by social determinants. For this reason, SHAPE projects represent the opportunity to develop learning strategies that impact the environment, and by implication, physical and mental health outcomes. A potential project could be based on climate change, health and wellness, by including elements of nature and sustainable foods in each of the observatories.

### 3.4. Design Thinking Models Applied to the Clinical Care of Women with Schizophrenia

Design thinking is a newly described process for dealing with complex problems whose causes are not fully known. It relies on empathy and working in collaborative multidisciplinary teams [65]. This approach prioritizes patient-oriented solutions and involves brainstorming, iteration and testing preliminary solutions.

Prior to the formation of observatories, we asked the following questions:

1. Who are the women we are trying to help?
2. What is the social and economic context in which they live?
3. What are these women’s topics of top concern?
4. Is there room for improvement in their health and social needs?
5. Can we find answers by talking to the women and their families, by referring to textbooks, consulting specialists and looking at the results of new research?

After generating as many solutions as possible, we designed the original five observatories.

### 3.5. Innovative Approaches Based on the Patient Education/Learning Project: Current and Future Perspectives

As patient education has been demonstrated to be effective in reducing risks, several learning strategies have been developed in our unit for women with schizophrenia.

Human learning through educational psychology has been widely recognized for many years [66]. Educational theories have moved from teacher-centered to student-centered approaches, and connectivism has been developed after discussions on traditional theories including behavioral, cognitivist and constructivist theories. Education in healthcare is based on patient care and working on everyday practical things [67]. In the context of mental health care, learning is a process of understanding new information in relation to what has already been learned, with the aim of changing behaviors and lifestyles [67]. Effective patient learning focuses on the concept of patient-centered care or patient engagement and should consider patients’ care needs. As our unit was designed as a result of the physical, mental and social needs of women with schizophrenia, the patient learning processes we have designed take into account the patients’ needs and the priorities of the patients.

The learning programs derived from the five working observatories for health are mainly based on learning/education strategies designed through Bloom’s Revised Taxonomy. This Revised Taxonomy has been considered one of the most relevant cognitive approaches to working on learning processes, including healthcare education. It has been already used in the development of active learning strategies including gamification [68]. In fact, Bloom’s Taxonomy has been considered an educational classification system to guide structured approaches to clinical reasoning [69].

Three main domains of an educational framework have been defined in Bloom’s Taxonomy: cognitive, affective and psychomotor learning [70]. In our learning project, we will focus on the cognitive domain, which includes six different levels or categories. The first category is called “to remember”, which means retrieving or recalling relevant knowledge from a certain topic. The second category or level is called “understand”. This consists of the determination, summary or comparison of knowledge or information with the previously learned information. The third level is called “to apply”, which means carrying out, executing or using the information to solve a given problem or situation. The fourth category or level is to “analyze”. This consists of organizing or attributing different parts of a structure. The fifth category is to “evaluate”, which leads the individual to make judgments based on the learned criteria or standards. The sixth category is “to create”, which means to generate or plan a novel strategy. The taxonomy uses these six cognitive action verbs within each of the six levels. Bloom’s Revised Taxonomy has been applied to the development of psychotherapeutic games with concrete learning objectives and can promote the construction of a psychiatric formulation or a learning process for patients [69].

#### 3.5.1. Learning Strategies in the Observatory for Substance Use Disorders

In the context of substance use disorders, Bloom’s Revised Taxonomy has already been applied to the development of courses on continuing education for healthcare professionals and students [70]. This study included the Substance Abuse Attitude Scale (SAAS) and the Liverpool Communication Skills Assessment Scale (LCSAS). We consider that this strategy may be useful for designing a course for women with schizophrenia with substance use disorder-related comorbidities and may be a useful framework strategy for a future specialized intervention in our unit. The main focus of the planned course should be early detection, prevention and accessibility to addiction services. The first level, “remember”, should be focused on the presentation of concepts of addictive behaviors and substances. The second level, “understand”, should be focused on the application of these concepts on the impact they have on physical health. The third level, “to apply”, is mainly focused on the use of prevention and management strategies to real-life circumstances. The concepts and situations will be applied to the social context. “To analyze” and to “evaluate” are the fourth and fifth levels, which are focused on the judgment of patients in group discussions. Women with schizophrenia should be capable of critically discussing the effects of benzodiazepine and alcohol consumption, as well as other substances, on their mental health and overall clinical prognosis. The patients may “create” a group of support oriented to substance use disorder-related comorbidity. This learning project is still in the planning phase.

#### 3.5.2. Learning Strategies in the Observatory for Somatic Morbi-Mortality

##### Physical Exercise

The observatory for somatic morbi-mortality aims to detect cardiovascular risk factors and to design and implement strategies to improve the health and quality of care of women with schizophrenia. The use of Bloom’s Taxonomy has been discussed with a focus on its implications on medical education in general [71]. Physical exercise recommendations seem to be effective in reducing cardiovascular risk factors in patients suffering from psychosis [72,73]. The vigilance team in this observatory has proposed a group of nutrition and physical exercise strategies. The co-participation of patients in the design of specialized interventions has been discussed. This group is starting in our unit; however, the inclusion of transformative approaches has been recently proposed. The first level should include activities focused on promoting the benefits and different types of exercise. Flipped-classroom strategies can be developed at this stage. The patients should briefly prepare the information to be shared with the other patients [74]. Understanding lifestyle benefits would be helpful for future stages. An analysis of menu suggestions would be helpful at this phase. The third phase, “to apply”, can focus on developing tasks that integrate the understood concepts to solve real-life problems. Problem-based learning strategies can be applied in this phase of the exercise group [75]. Analyzing all the information about the benefits of exercise can be discussed in the fourth phase (“analyze”). Cooperative techniques such as the jigsaw technique in cooperative learning methods can be used in the group phase [76]. The fifth phase of the group is focused on working on “evaluation” or critical judgement. The patients can evaluate their peers and propose solutions to problems that are proposed by the other members of the group. The sixth phase is based on “the creation” of calendars and exercise programs, as a result of the categorization of the information and the self-assessment carried out by the patients [77].

##### Dietary and Nutrition Recommendations

The application of Bloom’s Taxonomy to dietary education has been used for longer for patients with type 2 diabetes [78]. In patients with schizophrenia, the association between diet and psychosis, including aspects of nutritional deficiencies, have been extensively investigated [79,80,81]. In our unit, we plan a group of nutrition and diet recommendations according to the six levels of categories. For the “Remember or Knowledge” category, we propose expositive activities with the participation of patients to remember and increase knowledge about the five basic food groups: vegetables, fruits, dairy, starchy carbohydrates, oils, and spreads [79]. The “Understanding or Comprehension” level connects the current and previous knowledge about the nutrients contained in the five main foods. The third level is the “application” of the nutritional knowledge to modify the women’s existing diet and to adapt this behavior to real-life circumstances. The fourth level is based on “analysis”, which consists of interpreting food labels. The women may be asked to visit a supermarket to work on these topics. This includes meal planning according to their understanding of nutrients. The fifth category is the “evaluation” or interpretation of a diet in response to the individual results. Difficulties arise at this level. The sixth and the last level is the “creation” of a modified diet and the creation of new menus based on the knowledge and understanding of nutrition and its application to life circumstances and difficulties. This project of the learning group can be considered as an innovative project, initially reactive with the intention to be proactive, because the main aim of this observatory is to prevent somatic morbidities and mortality attributed to certain medical conditions [81].

#### 3.5.3. Learning Strategies in the Observatory for Social Exclusion and Discrimination

##### Transcultural Programs

The observatory for social exclusion and discrimination is currently centered on the improvement of clinical care for migrant women with schizophrenia [82,83,84]. A transformative innovation project is planned for this observatory because discriminated-against and socially excluded women should be part of the change and should be integrated in the design and implementation of intervention strategies. Cultural diversity programs for patients with schizophrenia are learning projects with a significant impact on health [85]. In recent years, several authors have reported that collaboration with patients may include cultural practices and beliefs to ensure adherence to treatment plans. However, most health education programs focus on the cultural competence of professionals and do little to involve patients in the learning and healthcare process [85,86]. As the patient education process has the aim of improving adherence to healthcare services, several authors have evaluated the effectiveness of early psychosis treatment in engaging immigrants. Ouellet-Plamondon et al. investigated the engagement in treatment and the medication compliance of immigrants and nonimmigrant patients in their first episode of psychosis who attended an early intervention service [87]. Medication adherence was found to be similar in both the immigrant and nonimmigrant groups, probably because this personalized service aims to provide better care specifically for these populations. The involvement of patients in the design of health education programs is important, as many barriers to accessing mental health services have been identified. Anti-stigma and anti-discrimination practices should consider patient involvement in campaigns and education programs.

##### Migration Programs

Immigrant patients are particularly at risk of discrimination [87]. Educational training strategies should include some aspects of Bloom’s Revised Taxonomy. For the first level, or “remember” category, we propose that patients should remember some concepts or knowledge about discrimination. The second level, the “understand” category, aims to improve the understanding of these concepts by integrating them with previous thoughts. The third level will be the “application” of concepts to real-life situations and social circumstances. The patients should propose anti-stigma interventions for or attitudes toward real problems. The fourth level, “the analysis”, leads the patients to interpret what they can really change and what they can propose to the healthcare system to enhance the social impact on their lives. The fifth level, the “evaluation”, refers to the participation of patients in the interpretation of the intervention’s results and the proposal of feedback to incorporate the results into practice. The sixth level, to “create”, means that migrant women with schizophrenia are active agents in the design and implementation of intervention strategies and are involved in design thinking. Currently, we are organizing a focus group of migrant women with schizophrenia to analyze barriers to the access and participation of these women in mental health services [83].

## 4. Conclusions

The Mutua Terrassa Functional Unit for Women with Schizophrenia was designed because of the observation that special clinics for women with schizophrenia, in contrast, for instance, to clinics for first-episode schizophrenia, did not exist [34]. Women with schizophrenia have clinical and social needs that differ, in many ways, from those of men. The many differences between men and women lend themselves to specific interventions as well as to specific learning objectives, both of which can be considered innovative. Healthcare projects are mainly formed by five original observatories or working groups (somatic morbi-mortality, hyperprolactinemia, substance use disorders, social exclusion and discrimination, and prescription and drug safety) [33]. The healthcare innovations incorporate reactive or proactive approaches (e.g., observatory for hyperprolactinemia) and transformative and incremental approaches (e.g., observatory for social exclusion and discrimination).

The learning or educational project is based on learning strategies considering cognitive factors inspired on the Revised Taxonomy of Bloom. To remember/know, to understand, to apply, to analyze, to evaluate, and to create specific interventions, are potentially more effective with the involvement of patients in the healthcare programs. This is an example of transformative approaches in the learning projects of the unit.

For the development of future similar clinics, we recommend the inclusion of peer-to-peer programs. We would recommend designing the components of a peer-to-peer training program for women with schizophrenia and to adequately plan the training logistics. Measurable outcomes may include variables assessing the impact of peer-to-peer-programs, for instance, on the access and adherence of women with schizophrenia to mental health services, the utilization of and satisfaction with services and the global functionality and well-being perception of patients. Our hypothesis, still to be tested, is that our innovations will improve patient satisfaction and overall outcomes and will result in forward-thinking clinicians.

## Figures and Tables

**Table 1:** Science and social/cultural corners of the Unit for Women with Schizophrenia.

**Science Corners**	
Clinical corner	Observatories of Health and Social Risk Factors
	Vigilance Teams or Monitoring Stations
	Specific Interventions
Research corner	Projects and Collaborations
**Social and Cultural Corners**	
Diversity corners	Cultural Awareness Program
	Transcultural Diversity Program
Cultural corners	Eating and Culture Corner

**Table 2:** The five original observatories of the Unit for Women with Schizophrenia.

Observatory	Objectives	New Preventive and Therapeutic Interventions	Evaluation Methods (Indicators: OI, PI)
Somatic morbi-mortality	Review all deaths and their pathogenesis Review medical comorbidities	1. Consultation with primary care/medical specialties 2. Physical exercise/dietary learning groups	PI: Evaluation of respiratory diseases; CVRF; screenings OI: Reduction in CVRF; increase in gynecological screenings
Hyperprolactinemia (HPRL)	Prevent and/or treat HPRL complications	Consultation with experts in neuroendocrinology	PI: Assessment of PRL levels and lowering strategies OI: Reduction in PRL levels
Substance use disorders	Prevent and/or treat comorbid substance use	Consultation with addiction experts	PI: Assessment of BZD and alcohol use OI: Reduction in BZD prescription and alcohol use
Social exclusion and discrimination	Detect, monitor and intervene in risk factors for discrimination, social isolation and acculturation difficulties	1. Cultural awareness programs for staff 2. Cultural diversity programs for patients	PI: Assessment of SDoH in migrant women OI: Increase in the access and participation of migrant women
Prescription and drug safety	Monitor drug safety and treatment response	1. Consultation with pharmacologists and pharmacogeneticists 2. Monitoring of drug levels and drug–drug interactions	PI: Assessment of antipsychotic combinations and risk of falls. OI: Reduction in antipsychotic combinations and percentage of falls.

Abbreviations: BZD, benzodiazepines; CVRF, cardiovascular risk factors; OI, outcome indicator; PI, process indicator; PRL, prolactin; SDoH, social determinants of health.

**Table 3:** Gathering of ideas, strategic planning, execution, feedback and iteration in healthcare and learning projects of the Unit of Women with Schizophrenia.

Definition	Phase 1	Phase 2	Phase 3	Phase 4
	Observation and Gathering of Ideas	Strategic Planning	Execution of Strategies	Feedback and Iteration and Scaling
Innovative Healthcare Project				
Aims	To explore health and social needs of women according to distinct needs	To plan feasible preventive interventions	To implement specific preventive strategies and to identify evaluation strategies (five observatories)	To receive feedback from patients, staff and consultants
Outcomes	To generate and select ideas that are evidence-based and feasible	To develop a calendar of implementation with specific goals for each stage	Evaluation of outcomes of pilot interventions	Iteration and correction of interventions
Innovative Learning Project				
Aims	To explore learning needs of patients with regard to healthcare	To design learning activities focused on topics of concern using active learning strategies	To execute learning activities and evaluation of learning outcomes	To receive feedback from patients and design needed changes in learning activities
Outcomes	To analyze learning needs implicated in healthcare processes of somatic morbi-mortality, hyperprolactinemia, substance use disorders, social exclusion/discrimination and drug safety	To prepare learning materials in the context of the Revised Taxonomy of Bloom	To use technology to present prepared reviews, charts, illustrations, videos	To improve classroom materials and to propose new case studies to cover complex situations
Future perspectives including technological techniques	Generation of innovative ideas by using electronic boards Selection of ideas by using specific techniques such as empathy maps (collaborative visualization)	To create a platform of contents, classified into topics of concern	To build short videos or micro-videos of relevant themes to be presented in hybrid presentations	To include new videos and modify contents of the platform To use discussion forum to share knowledge and maintain upkeep

## Data Availability

The data presented in this review are available upon request from the corresponding author.

## References

[1] Li X., Zhou W., Yi Z. (2022). A glimpse of gender differences in schizophrenia. Gen. Psychiatry.

[2] Szeliga A., Stefanowski B., Meczekalski B., Snopek M., Kostrzak A., Smolarczyk R., Bala G., Duszewska A., Smolarczyk K., Maciejewska-Jeske M. (2021). Menopause in women with schizophrenia, schizoaffective disorder and bipolar disorder. Maturitas.

[3] Ochoa S., Usall J., Cobo J., Labad X., Kulkarni J. (2012). Gender differences in schizophrenia and first-episode psychosis: A comprehensive literature review. Schizophr. Res. Treatment..

[4] Seeman M.V. (2023). Schizophrenia in Women: Clinical Considerations. Psychiatr. Clin. N. Am..

[5] Mu E., Gurvich C., Kulkarni J. (2024). Estrogen and psychosis—A review and future directions. Arch. Women’s Ment. Health.

[6] Tiwari S., Prasad R., Wanjari M.B., Sharma R. (2023). Understanding the Impact of Menopause on Women With Schizophrenia-Spectrum Disorders: A Comprehensive Review. Cureus.

[7] Ferrara M., Curtarello E.M.A., Gentili E., Domenicano I., Vecchioni L., Zese R., Alberti M., Franchini G., Sorio C., Benini L. (2024). Sex differences in schizophrenia-spectrum diagnoses: Results from a 30-year health record registry. Arch. Women’s Ment. Health.

[8] Giordano G.M., Bucci P., Mucci A., Pezzella P., Galderisi S. (2021). Gender Differences in Clinical and Psychosocial Features Among Persons With Schizophrenia: A Mini Review. Front. Psychiatry.

[9] González-Rodríguez A., Seeman M.V., Natividad M., Barrio P., Román E., Balagué A., Paolini J.P., Monreal J.A. (2023). Review of Male and Female Care Needs in Schizophrenia: A New Specialized Clinical Unit for Women. Women.

[10] Kulkarni J., Fitzgerald P., Seeman M.V., Castle D.J., Copolov D.L., Wykes T., Mueser K.T. (2012). Clinical needs of women with schizophrenia. Pharmacological and Psychosocial Treatments in Schizophrenia.

[11] Correll C.U., Solmi M., Croatto G., Schneider L.K., Rohani-Montez S.C., Fairley L., Smith N., Bitter I., Gorwood P., Taipale H. (2022). Mortality in people with schizophrenia: A systematic review and meta-analysis of relative risk and aggravating or attenuating factors. World Psychiatry.

[12] Lu C., Jin D., Palmer N., Fox K., Kohane I.S., Smoller J.W., Yu K.H. (2022). Large-scale real-world data analysis identifies comorbidity patterns in schizophrenia. Transl. Psychiatry.

[13] Hodgson R., Wildgust H.J., Bushe C.J. (2010). Cancer and schizophrenia: Is there a paradox?. J. Psychopharmacol..

[14] Mucheru D., Hanlon M.C., Campbell L.E., McEvoy M., MacDonald-Wicks L. (2018). Cardiovascular disease lifestyle risk factors in people with psychosis: A cross-sectional study. BMC. Public Health.

[15] Drevinskaite M., Kaceniene A., Patasius A., Stukas R., Germanavicius A., Miseikyte E., Urbonas V., Smailyte G. (2024). Cancer mortality and morbidity among patients with schizophrenia: A hospital-based cohort study, 1992–2020. Acta. Psychiatr. Scand..

[16] O’Neill B., Yusuf A., Lofters A., Huang A., Ekeleme N., Kiran T., Greiver M., Sullivan F., Kurdyak P. (2023). Breast Cancer Screening Among Females With and Without Schizophrenia. JAMA. Netw. Open.

[17] Malaspina D. (2023). What social determinants can tell us about schizophrenia. Schizophr. Res..

[18] Jeste D.V., Malaspina D., Bagot K., Cole S., Dickerson F., Dilmore A., Ford C.L., Karcher N.R., Luby J., Rajoo T. (2023). Review of major social determinants of health in schizophrenia-spectrum psychotic disorders: III. Biology. Schizophr. Bull..

[19] Jester D.J., Thomas M.L., Sturm E.T., Harvey P.D., Keshavan M., Davis B.J., Saxena S., Tampi R., Leutwyler H., Compton M.T. (2023). Review of major social determinants of health in schizophrenia-spectrum psychotic disorders: I. Clinical outcomes. Schizophr. Bull..

[20] Roberts L.W., Warner T.D., Nguyen K.P., Geppert C.M., Rogers M.K., Roberts B.B. (2003). Schizophrenia patients’ and psychiatrists’ perspectives on ethical aspects of symptom re-emergence during psychopharmacological research participation. Psychopharmacology.

[21] Walker V.G., Harrison T.C. (2024). Life Course Perspectives of Aging With Schizophrenia Spectrum Disorders in Psychiatric and Long-Term Care Facilities. Gerontologist.

[22] Ii S.S., Fitzgerald L., Morys-Carter M.M., Davie N.L., Barker R. (2018). Knowledge translation in tri-sectoral collaborations: An exploration of perceptions of academia, industry and healthcare collaborations in innovation adoption. Health Policy.

[23] Chivilgina O., Elger B.S., Jotterand F. (2021). Digital Technologies for Schizophrenia Management: A Descriptive Review. Sci. Eng. Ethics.

[24] Schipper S. (2020). Innovative collaborations are improving mental health care. Can. Fam. Phys..

[25] Kelly C.J., Young A.J. (2017). Promoting innovation in healthcare. Future Health J..

[26] Herodotou C., Sharples M., Gaved M., Kukulska-Hulme A., Rienties B., Scanlon E., Whitelock D. (2019). Innovative pedagogies of the future: An evidence-based selection. Front. Educ..

[27] Češková E., Šilhán P. (2021). From personalized medicine to precision psychiatry?. Neuropsychiatr. Dis. Treat..

[28] Kiely K.M., Brady B., Byles J. (2019). Gender, mental health and ageing. Maturitas.

[29] Kulkarni J. (2023). Estrogen—A key neurosteroid in the understanding and treatment of mental illness in women. Psychiatry Res..

[30] Riecher-Rössler A. (2017). Oestrogens, prolactin, hypothalamic-pituitary-gonadal axis, and schizophrenic psychoses. Lancet Psychiatry.

[31] Brand B.A., de Boer J.N., Dazzan P., Sommer I.E. (2022). Towards better care for women with schizophrenia-spectrum disorders. Lancet Psychiatry.

[32] Sommer I.E., Brand B.A., Gangadin S., Tanskanen A., Tiihonen J., Taipale H. (2023). Women with schizophrenia-spectrum disorders after menopause: A vulnerable group for relapse. Schizophr. Bull..

[33] Natividad M., Seeman M.V., Paolini J.P., Balagué A., Román E., Bagué N., Izquierdo E., Salvador M., Vallet A., Pérez A. (2023). Monitoring the effectiveness of treatment in women with schizophrenia: New specialized cooperative approaches. Brain Sci..

[34] Seeman M.V., Cohen R. (1998). A service for women with schizophrenia. Psychiatr. Serv..

[35] Paterick T.E., Patel N., Tajik A.J., Chandrasekaran K. (2017). Improving health outcomes through patient education and partnerships with patients. Bayl. Univ. Med. Cent. Proc..

[36] Bhattad P.B., Pacifico L. (2022). Empowering Patients: Promoting Patient Education and Health Literacy. Cureus.

[37] Lang J.P., Jurado N., Herdt C., Sauvanaud F., Lalanne Tongio L. (2019). Education care in patients with psychiatric disorders in France: Psychoeducation or therapeutic patient education?. Rev. Epidemiol. Sante Publique.

[38] Hempler N.F., Pals R.A.S., Pedersbæk L., DeCosta P. (2018). Barriers and facilitators of effective health education targeting people with mental illness: A theory-based ethnographic study. BMC Psychiatry.

[39] Maguire T.K., Chen J. (2023). Innovation in Mental Health Care: Expanding Collaborations and Building Digital Tools. Am. J. Geriatr. Psychiatry.

[40] Allen C., Metternicht G., Eiedmann T. (2018). Initial progress in implementing the Sustainable Development Goals (SDGs): A review of evidence from countries. Sustain. Sci..

[41] Edinoff A.N., Silverblatt N.S., Vervaeke H.E., Horton C.C., Girma E., Kaye A.D., Kaye A., Kaye J.S., Garcia A.J., Neuchat E.E. (2021). Hyperprolactinemia, clinical considerations, and infertility in women on antipsychotic medications. Psychopharmacol. Bull..

[42] Taipale H., Solmi M., Lähteenvuo M., Tanskanen A., Correll C.U., Tiihonen J. (2021). Antipsychotic use and risk of breast cancer in women with schizophrenia: A nationwide nested case-control study in Finland. Lancet Psychiatry.

[43] Brand B.A., Willemse E.J.M., Hamers I.M.H., Sommer I.E. (2023). Evidence-based recommendations for the pharmacological treatment of women with schizophrenia spectrum disorders. Curr. Psychiatry Rep..

[44] Casanovas F., Fonseca F., Mané A. (2023). Substance use specificities in women with psychosis: A critical review. Curr. Neuropharmacol..

[45] Aakre J.M., Brown C.H., Benson K.M., Drapalski A.L., Gearon J.S. (2014). Trauma exposure and PTSD in women with schizophrenia and coexisting substance use disorders: Comparisons to women with severe depression and substance use disorders. Psychiatry Res..

[46] Vallejo-Martín M., Sánchez Sancha A., Canto J.M. (2021). Refugee women with a history of trauma: Gender vulnerability in relation to post-traumatic stress disorder. Int. J. Environ. Res. Public Health.

[47] Vu A., Adam A., Wirtz A., Pham K., Rubenstein L., Glass N., Beyrer C., Singh S. (2014). The prevalence of sexual violence among female refugees in complex humanitarian emergencies: A systematic review and meta-analysis. PLoS Curr..

[48] Lichtenstein A.H., Berger A., Cheng M.J. (2017). Definitions of healing and healing interventions across different cultures. Ann. Palliat. Med..

[49] Schilder K., Tomov T., Mladenova M., Mayeya J., Jenkins R., Gulbinat W., Manderscheid R., Baingana F., Whiteford H., Khandelval S. (2004). The appropriateness and use of focus group methodology across international mental health communities. Int. Rev. Psychiatry.

[50] Seeman M.V. (2020). Men and women respond differently to antipsychotic drugs. Neuropharmacology.

[51] González-Rodríguez A., Guàrdia A., Álvarez Pedrero A., Betriu M., Cobo J., Acebillo S., Monreal J.A., Seeman M.V., Palao D., Labad J. (2020). Women with Schizophrenia over the life span: Health promotion, treatment and outcomes. Int. J. Environ. Res. Public Health.

[52] Waldman S.A., Terzic A. (2019). Health care evolves from reactive to proactive. Clin. Pharmacol. Ther..

[53] Castle D., Li A. (2023). Physical health monitoring for people with schizophrenia. Aust. Prescr..

[54] Danda D. (2013). Next generation evidence-based medicine: Individualised, personalised and humanised. Int. J. Rheum. Dis..

[55] Thijssen S.V., Jacobs M.J.G., Swart R.R., Heising L., Ou C.X.J., Roumen C. (2021). The barriers and facilitators of radical innovation implementation in secondary healthcare: A systematic review. J. Health Organ. Manag..

[56] Bova R., Lusardi R. (2022). The mental health of migrant people: Western medical systems facing a globalized society. Sociol. Compass.

[57] Seeman M.V. (2010). Raves, psychosis, and spirit healing. Transcult. Psychiatry.

[58] Greben S.E., Seeman M.V. (1994). Learning from the patient. Hum. Med..

[59] Lee Y.S.H., Grob R., Nembhard I., Shaller D., Schlesinger M. (2024). Leveraging Patients’ creative ideas for innovation in health care. Milbank Q..

[60] Zhan Z., Hu Q., Liu X., Wang S. (2023). STEAM Education and the innovative pedagogies in the intelligence era. Appl. Sci..

[61] Masters J.C., Kane M.F., Pike M.E. (2014). The suitcase simulation: An effective and inexpensive psychiatric nursing teaching activity. J. Psychosoc. Nurs. Ment. Health Serv..

[62] Morgan Jones M., Abrams D., Lahiri A. (2020). Shape the Future: How the social sciences, humanities and the arts can SHAPE a positive post-pandemic future for peoples, economies and environments. J. Br. Acad..

[63] Isabel F., Yiangou D., Ang C., Parkinson S., Guthrie S. (2023). Understanding Social Sciences, Humanities and Arts for People and the Economy (SHAPE) R&D in the UK and Internationally.

[64] Onaolapo O.J., Onaolapo A.Y. (2021). Nutrition, nutritional deficiencies, and schizophrenia: An association worthy of constant reassessment. World J. Clin. Cases.

[65] Altman M., Huang T.T.K., Breland J.Y. (2018). Design thinking in health care. Prev. Chronic Dis..

[66] Lockey A., Conaghan P., Bland A., Astin F. (2020). Educational theory and its application to advanced life support courses: A narrative review. Resusc. Plus.

[67] Brown J. (2020). Using Learning Theory to Shape Learning Experiences in Health Care Education: Not Scary at All. New Dir. Teach. Learn.

[68] Haring P., Warmelink H., Valente M., Roth C. (2018). Using the Revised Bloom Taxonomy to Analyze Psychotherapeutic Games. Int. J. Games Technol..

[69] de Beer W.A. (2017). Original opinion: The use of Bloom’s Taxonomy to teach and assess the skill of the psychiatric formulation during vocational training. Australas. Psychiatry.

[70] Muzyk A.J., Tew C., Thomas-Fannin A., Dayal S., Maeda R., Schramm-Sapyta N., Andolsek K., Holmer S. (2018). Utilizing Bloom’s taxonomy to design a substance use disorders course for health professions students. Subst. Abus..

[71] Miller D.A., Sadler J.Z., Mohl P.C., Melchiode G.A. (1991). The cognitive context of examinations in psychiatry using Bloom’s taxonomy. Med. Educ..

[72] Sharma A., Madaan V., Petty F.D. (2006). Exercise for mental health. Prim. Care Companion J. Clin. Psychiatry.

[73] Ho P.A., Dahle D.N., Noordsy D.L. (2018). Why Do People With Schizophrenia Exercise? A Mixed Methods Analysis Among Community Dwelling Regular Exercisers. Front. Psychiatry.

[74] Ji M., Luo Z., Feng D., Xiang Y., Xu J. (2022). Short- and Long-Term Influences of Flipped Classroom Teaching in Physiology Course on Medical Students’ Learning Effectiveness. Front. Public Health.

[75] Kalu F., Wolsey C., Enghiad P. (2023). Undergraduate nursing students’ perceptions of active learning strategies: A focus group study. Nurse Educ. Today.

[76] Goolsarran N., Hamo C.E., Lu W.H. (2020). Using the jigsaw technique to teach patient safety. Med. Educ. Online.

[77] Schiel K.Z., Everard K.M. (2021). Active Learning Versus Traditional Teaching Methods in the Family Medicine Clerkship. Fam. Med..

[78] Arana M.A., Valderas J.M., Solomon J. (2019). Being tested but not educated—A qualitative focus group study exploring patients’ perceptions of diabetic dietary advice. BMC Fam. Pract..

[79] Aucoin M., LaChance L., Cooley K., Kidd S. (2020). Diet and Psychosis: A Scoping Review. Neuropsychobiology.

[80] Morris A., Reed T., McBride G., Chen J. (2024). Dietary interventions to improve metabolic health in schizophrenia: A systematic literature review of systematic reviews. Schizophr. Res..

[81] van Zonneveld S.M., Haarman B.C.M., van den Oever E.J., Nuninga J.O., Sommer I.E.C. (2022). Unhealthy diet in schizophrenia spectrum disorders. Curr. Opin. Psychiatry.

[82] Morgan C., Knowles G., Hutchinson G. (2019). Migration, ethnicity and psychoses: Evidence, models and future directions. World Psychiatry.

[83] Trabsa A., Casanovas F., Pérez V., Moreno A., Amann B., Mané A. (2024). Comparison of male and female non-refugee immigrants with psychosis: Clinical, sociodemographic, and migration-related differences and impact on stress. Arch. Women’s Ment. Health.

[84] Hollander A.C., Dal H., Lewis G., Magnusson C., Kirkbride J.B., Dalman C. (2016). Refugee migration and risk of schizophrenia and other non-affective psychoses: Cohort study of 1.3 million people in Sweden. BMJ.

[85] Price J.L., Cordell B. (1994). Cultural diversity and patient teaching. J. Contin. Educ. Nurs..

[86] Spinner J.R., Haynes E., Nunez C., Baskerville S., Bravo K., Araojo R.R. (2021). Enhancing FDA’s Reach to Minorities and Under-Represented Groups through Training: Developing Culturally Competent Health Education Materials. J. Prim. Care Community Health.

[87] Ouellet-Plamondon C., Rousseau C., Nicole L., Abdel-Bakmi A. (2015). Engaging Immigrants in Early Psychosis Treatment: A Clinical Challenge. Psychiatr. Serv..

